# Ethnocentrism and place identity in the consumption of local products

**DOI:** 10.1016/j.heliyon.2024.e31602

**Published:** 2024-05-21

**Authors:** Edgar J. Sabina del Castillo, Ricardo J. Díaz Armas, Desiderio Gutiérrez Taño

**Affiliations:** University of La Laguna Department of Business Management and Economic History Camino La Hornera, 37. Faculty of Economics, Business and Tourism, P.O. Box 456, 38200, San Cristóbal de La Laguna Canary Islands, Spain

**Keywords:** Attitude towards local products, Consumer ethnocentrism, Place identity, Moderated mediation model

## Abstract

Research on the consumption of local products is essential to promote sustainability, boost local economies, and preserve cultural identity. Although a positive relationship has been demonstrated between attitude towards local products and consumption determinants, the role of the former as a mediator has not been sufficiently explored. This study examines how the attitude towards local products mediates between consumer ethnocentrism and consumption intention, as well as between place identity and consumption intention. A total of 1325 wine and cheese consumers in the Canary Islands were surveyed using a moderated mediation model, applying PLS-SEM. The results indicate that attitude towards local products mediates the aforementioned relationships but does not moderate them according to the type of local product. Consequently, marketing strategies should focus on the emotional and cultural connection that consumers establish with local products, highlighting their value in terms of identity and belonging.

## 1 Introduction

In the era of globalisation, the consumption of local products emerges as a key factor in promoting global sustainability, strengthening regional economies, and preserving cultural identities [[1], [2], [3]]. This trend towards localisation has also generated positive environmental impacts by reducing fuel use and minimising the environmental impact linked to global food transport [[4], [5], [6]]. This paradigm shift becomes even more relevant when we consider that exports have been identified as a crucial factor in environmental pollution patterns [7]. Both energy and natural resources used in the various stages of the production of internationally traded goods can cause environmental pollution [[8], [9], [10]]. Moreover, unsustainable consumption and production patterns, particularly prevalent in industrialised economies, represent one of the main drivers of continued environmental degradation on a global scale [11]. Consequently, there is a clear need for governments to implement policies geared towards sustainable economic growth and to invest more in renewable energy, thereby strengthening resilience to protect people's health [12]. These actions should be grounded in ESG (Environment, Society and Corporate Governance) principles and aligned with the Sustainable Development Goals (SDGs) of the United Nations 2030 Agenda [13]. In particular, SDG 12 highlights the imperative need to ensure sustainable consumption and production patterns [14], thus positioning the promotion of local products as an essential component for the achievement of these global goals.

On the other hand, the COVID-19 pandemic, which triggered an unprecedented crisis in various sectors of our society [15], evidenced a significant change in purchasing and consumption habits [16]. This extreme situation highlighted the close relationship between our health, the environment and the economy [17]. It also underlined the importance of strengthening our local food systems and ensuring food security through shorter supply chains [[18], [19], [20]]. The valorisation of our local production and local markets [21,22] emerged as a priority, generating a renewed interest among consumers to know the origin and characteristics of the products they buy [[16], [23]].

In this context, normative factors such as consumer ethnocentrism and affective factors such as place identity emerge as key pillars influencing consumer preferences for local products [24,25]. The direct relationship between consumer ethnocentrism and positive attitudes towards local products, as well as the connection between place identity and the selection of these products, underlines their determinant role [[1], [26], [27]].

Despite the undeniable relevance of this topic, there are significant gaps in the current literature related to these variables. In particular, it has not been sufficiently explored whether the relationship between these variables and the consumption of local products may be mediated by attitudes towards these products. Furthermore, current studies have paid little attention to specific types of products, a crucial shortcoming given the complexity of food selection [28]. To address these shortcomings, our study analyses the mediating role of attitudes towards local products in the relationships between consumer ethnocentrism, place identity and consumption intention. Furthermore, it examines whether there is a moderating effect on these relationships according to the type of product, using two specific local products: wine and cheese.

There are several reasons for choosing these two products: firstly, both wine and cheese have deep roots in the culture and traditions of the regions where they are produced, giving them significant relevance in terms of identity [29,30]; secondly, their consumption is widespread across diverse cultures worldwide [31,32], which facilitates the comparison and generalisation of our findings; similarly, both products have a relevant economic impact in multiple regions, contributing to the generation of employment and the promotion of tourism, which underlines their importance in the dynamisation of local economies [33,34]; and, finally, a significant number of these products bear Protected Designation of Origin (PDO) seals. These certifications not only provide details about their provenance and guarantee a quality influenced by factors such as soil, climate or regional production experience but also contribute to the preservation of traditional cultural values [35].

To achieve our research objectives, self-administered online surveys were administered to wine and cheese consumers living in the Canary Islands (Spain) in 2020, resulting in a sample of 1325 people. The Partial Least Squares Structural Equation Modelling technique, otherwise known as PLS-SEM, was used to analyse the proposed conceptual moderated mediation model and test the hypotheses.

The Canary Islands were chosen as the setting for our study due to their excellent tradition of wine and cheese production. Canarian wines stand out for their quality and authenticity thanks to volcanic soils, microclimates and grape varieties that do not exist anywhere else in the world. The islands produce on average 10 million litres of wine a year [36]. At the same time, this region has the highest cheese consumption per capita in Spain, producing around 17,000 tonnes a year and boasting multiple international awards [37,38]. Furthermore, the geographical location of the archipelago, its relative isolation and its condition of being an ultra-peripheral region of the European Union, underline how important supporting local products is for its economy [39].

In the following sections, we will present a detailed literature review, followed by a description of the methodology used and the main results of our study. Finally, we will analyse the results of previous research, examine the practical implications derived from the findings and present the most relevant conclusions from our study.

## 2 Literature review

### 2.1 Theoretical framework

The scientific literature provides several theoretical approaches, widely recognised as explanatory models enabling us to understand consumption behaviour related to local products. The Theory of Planned Behaviour (TPB) [40] stands out as one of the most widely used in this field [[41], [42], [43], [44], [45], [46], [47]]. Within this framework, attitude emerges as the main determinant of intention to engage in specific behaviours [40], supported by extensive studies on consumption behaviour concerning local products [[48], [49], [50]].

In parallel, additional research in this discipline has incorporated variables into the original TPB model to address the complexity intrinsic to personal behaviour [51,52] or has relied on the Social Identity Theory [53,54] to explain consumer behaviour [[27], [55]]. The latter theory postulates that people tend to classify themselves and others into social groups, which influences their perception and judgement of themselves and others. This phenomenon also affects consumption choices as they become a means of building and maintaining self-esteem by conforming to the norms and values associated with those social groups [53,54].

Our study presents a conceptual moderated mediation model, based on the above-mentioned theoretical frameworks. Specifically, we investigated whether attitude towards local products acts as a mediating variable between consumer ethnocentrism (independent variable) and intention to consume local products (dependent variable). We also analysed whether said attitude plays a mediating role in the relationship between place identity (independent variable) and intention to consume local products (dependent variable). Furthermore, we aimed to determine whether the type of local product (be it wine or cheese) had a moderating effect on these relationships; that is, whether the intensity of the relationships varies according to the local product in question.

Mediation and moderation are emerging as fundamental concepts in the analysis of relationships between different variables. These concepts provide theoretical frameworks that facilitate the interpretation and understanding of the complex relationships between variables [56]. Mediation refers to the process through which a mediating variable explains the relationship between an independent variable and a dependent variable [57]. In other words, mediation arises when a third variable helps us to understand how or why two variables are related. The mediating variable can be considered an intervening or facilitating variable [58]. Moderation is related to a variable that modifies the strength or direction of the relationship between two variables [57]. Moderation suggests that the effects of an independent variable on a dependent variable may vary depending on the levels of another moderating variable [56]. This implies that the relationship between the variables is not constant but depends on certain contextual factors or specific conditions. Recently, moderating variables have become more relevant in the prediction of consumer behaviour as they specify the type of link that exists between independent and dependent variables [[59], [60], [61]].

### 2.2 Consumer ethnocentrism

Ethnocentrism is a sociological concept that describes a feeling or perspective whereby one's own culture is valued and considered to be better than others [62]. This idea has been adapted and expanded upon in the sphere of marketing and consumer behaviour, giving rise to ‘consumer ethnocentrism’. This term reflects consumers' tendencies to prefer products from their own culture or country over imported products, based on the belief that they are of a higher quality and more authentic or ethical than foreign products, which may influence purchase and consumption decisions [63]. However, ethnocentrism has evolved towards a deeper integration with other cultures, which is reflected by an attitude that combines a renewed appreciation of one's own culture and a genuine respect and openness towards other cultures. Instead of considering one's own culture as superior, cultural diversity is appreciated and celebrated, thus encouraging integration and cooperation between different cultures [64].

Consumers with a high level of ethnocentrism show a clear predilection for national products and are opposed to foreign brands and products [[65], [66], [67]]. This reticence may be due to nationalism, the perception of a negative impact on the local economy, anti-patriotic considerations, or even stereotypes or prejudices towards the product's country of origin [68,69]. This inclination is not only limited to national purchases but also extends to decisions to purchase local products [70]. An ethnocentric consumer tends to prefer local products over national ones and, in turn, prefers national products over imported ones [71]. It should also be noted that the feeling of ethnocentrism is not necessarily continuous or constant in the consumer, but may increase in certain contexts or adverse situations, such as a pandemic or war, driving the preference for local products such as wine [[43], [72]].

Numerous studies support the existence of a positive relationship between consumer ethnocentrism and attitude towards local products, including wine and cheese from specific regions [[26], [43], [73], [74], [75], [76], [77]]. Similarly, a positive link has been identified between consumer ethnocentrism and intention to consume said local products [78,79]. However, there is a lack of research that looks into the effect of consumer ethnocentrism on decisions related to specific food products [80]. Therefore, we propose the following hypotheses.

Hypothesis Ha1 (Ha1) There is a significant positive relationship between consumer ethnocentrism and attitude towards local products.

Hypothesis H1c’ (H1c′) There is a significant positive relationship between consumer ethnocentrism and intention to consume local products.

### 2.3 Place identity

Place identity is conceptualised as the part of an individual's personal identity that is formed by their experiences linked to specific places [81]. In other words, it reflects the emotional link and sense of belonging that people feel towards a particular place [82]. How long an individual stays in a place [83] and significant experiences there, whether positive or negative, may strengthen this link [84].

Place identity is not only formed based on direct experiences and memories associated with a particular place [85] but also through the relationships and communities that are built there [86]. The traditions, values and beliefs that are typical of a place, and which people adopt as part of their identity, are also fundamental in this process [87]. Nevertheless, in an era of globalisation, in which it is common for people to live in several different places throughout their lifetime, this dimension may acquire nuances and complexities that give rise to hybrid or multifaceted identities [88].

From a broader perspective, place identity can also be analysed from the point of view of national identity, which highlights the importance and value that individuals give to their sense of belonging to a nation [89,90]. This approach includes aspects such as belonging, responsibility, awareness, pride, and loyalty towards the nation [89]. Analysis of national product purchase decisions has revealed that national identity can have a positive effect [91], not due to the rejection of other countries, but because of a strong sense of belonging and loyalty towards one's own country [92]. In some cases, national identity can even lead consumers to prioritise the interests of the nation over their own [93]. Other authors have studied place identity taking into account the place attachment theory [94] or even as a dimension of place attachment that can significantly influence the intention to repurchase GI agricultural products [95].

Several authors back the idea that consumers with a pronounced place identity, whether related to their place of residence or country, show a preference towards products from that geographical area [26,27]. For example, research relating to specific products, such as local wine or cheese, suggests that consumers with a strong place identity tend to value these products more if they are produced in their region or country [[30], [97]]. Based on these considerations, we put forward the following hypotheses.

Hypothesis Ha2 (Ha2) There is a significant positive relationship between place identity and attitude towards local products.

Hypothesis H2c’ (H2c′) There is a significant positive relationship between place identity and intention to consume local products.

### 2.4 Attitude towards local products

Attitudes, conceived to be positive or negative opinions that an individual holds towards a range of stimuli such as people, events and products, play a crucial role in determining purchase intentions [98]. In the context of Ajzen's Theory of Planned Behaviour [40], these attitudes present themselves as essential deciding factors in the intention to carry out a specific behaviour as they reflect the individual assessment of said action. This implies that favourable or unfavourable attitudes towards a certain behaviour may have a significant relationship with intentions to engage in or avoid said behaviour. However, attitudes are dynamic and evolve with the perception of positive or negative aspects. This does not mean that a new attitude replaces the previous one; an educated predisposition is simply generated from accumulated perceptions and beliefs. Therefore, a person may have two attitudes towards the same stimulus at the same time: one that is implicit, created automatically and unconsciously based on past experiences; and one that is explicit, arising from a logical, reflexive, conscious process [99].

The relationship between attitude and behaviour is particularly evident in local products. Multiple studies [[50], [100], [101], [102]] have demonstrated a significant positive relationship between attitude towards local products and intention to consume them. For example, in sectors such as wine-making and cheese production, it has been revealed that a positive attitude towards local wine or cheese implies a greater predisposition to consume them [[103], [104], [105]]. The COVID-19 pandemic has added a further nuance since the changes in consumption patterns resulting from the crisis have led to a more favourable attitude towards local products, as several authors demonstrate [[25], [106], [107]]. In this context, taking into account the evidence of the above-mentioned studies, such as those of Skallerud & Wien [50] and Zhang et al. [108], we established the following hypothesis.

Hypothesis Hb (Hb) There is a significant positive relationship between attitude towards local products and intention to consume them.

In a large number of studies related to consumer behaviour, attitude towards products has been presented as a mediating variable. Many authors have demonstrated its relevance as a mediator between different variables [[109], [110], [111], [112]], highlighting the importance of considering attitude towards products as a key mediating element in certain relationships and contexts.

For example, in a study by Camacho et al. [113], attitude was found to play a mediating role between xenocentrism (opposing attitude to ethnocentrism) and purchase intention regarding imported products in Colombia. Similarly, Jianlin et al. [74] confirmed that attitude towards domestic products mediated the relationship between consumer ethnocentrism and purchase intention in China. By the same token, Chaturvedi et al. [65] validated that attitude mediated the connection between consumer ethnocentrism and intention to purchase locally produced organic food. In the wine domain, Maksan et al. [75] found that attitudes played a mediating role in the relationship between consumer ethnocentrism and intention to buy Croatian wine. Taking these results into consideration, we formulated the following hypothesis.

Mediation hypothesis H1 (H1med) *Attitude towards local products mediates the relationship between consumer ethnocentrism and intention to consume local products.*

As mentioned previously, the literature in the field of consumer behaviour has examined in detail the direct relationship between place identity and intention to consume local products [26,27], as well as the direct relationship between attitude towards said products and intention to consume them [[48], [49], [50], [74]]. Nevertheless, we have noticed that there is a significant lack of studies that consider attitude towards local products as a mediating variable between place identity and intention to consume local products. This gap in the literature provides an opportunity to conduct a more in-depth analysis and obtain a more detailed understanding of the factors that drive consumer decisions related to local products. Therefore, in this study, we have proposed the following hypothesis.

Mediation hypothesis H2 (H2med) *Attitude towards local products mediates the relationship between place identity and intention to consume local products.*

### 2.5 Research questions

Scientific research essentially seeks to expand the boundaries of knowledge by exploring and analysing phenomena that are not yet fully understood. In many cases, when the field is known and the relationships between variables have been previously established, it is possible to form clear, direct hypotheses that guide the study. However, in less explored areas or when faced with complex, multifaceted phenomena, the direct formulation of hypotheses may be premature. In such circumstances, it is more appropriate to address the research via research questions that allow for an open, exploratory approach, providing the necessary flexibility to discover and understand non-evident or unexpected relationships between variables.

This research ventures into a relatively unexplored field by examining how different types of local products, such as wine and cheese, can moderate the relationships between consumer ethnocentrism, place identity, attitude towards products and consumption intention. Given that local products may carry unique sensory, historical and cultural connotations [105], it is logical to assume that not all local products will have the same effect on consumer attitudes and behaviour.

Previous studies have shown that consumer behaviour may vary according to the type of product [[114], [115], [116], [117]]. However, there is very little research that addresses consumer preferences towards local foods and considers more than one product, although specific differences have been found based on the individual products studied [[116], [118], [119], [120], [121], [122], [123]]. Furthermore, there are discrepancies in the conclusions associated with consumer ethnocentrism [78,79] and place identity [124,125]. Given this situation and to avoid hurried assumptions within the scope of this study, we opted to formulate the following research questions and sub-questions.

Research question 1 (RQ1) *Does the type of local product moderate the mediating relationships proposed between consumer ethnocentrism and intention to consume local products through attitude towards local products? More specifically:*

RQ1a: Are there differences according to the type of local product in the relationships between consumer ethnocentrism and attitude towards local products?
RQ1b: Are there differences according to the type of local product in the relationships between consumer ethnocentrism and intention to consume local products?

Research question 2 (RQ2) *Does the type of local product moderate the mediating relationships proposed between place identity and intention to consume local products through attitude towards local products? More specifically:*

RQ2a: Are there differences according to the type of local product in the relationships between place identity and attitude towards local products?
RQ2b: Are there differences according to the type of local product in the relationships between place identity and intention to consume local products?

Research question 3 (RQ3) *Are there differences according to the type of local product in the relationships between attitude towards local products and intention to consume them?*

### 2.6 Proposed theoretical moderated mediation model

**Fig. 1:**
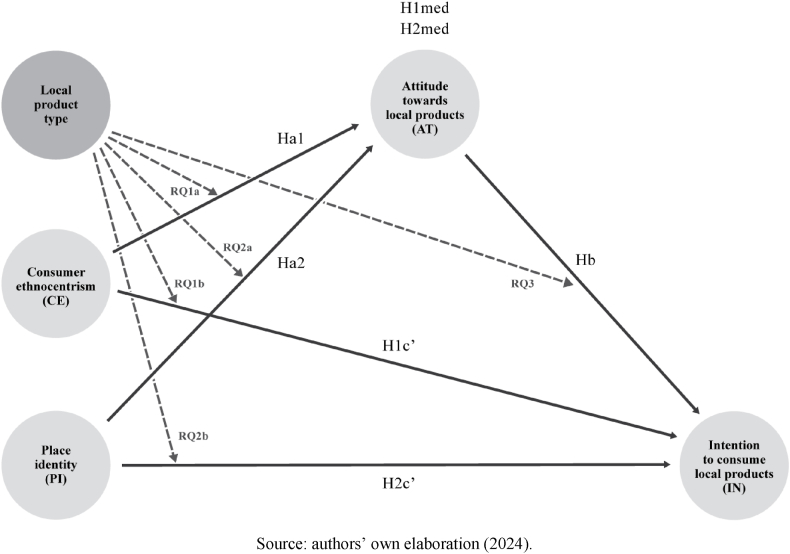
Proposed theoretical moderated mediation model.

Source: authors' own elaboration (2024).

The theoretical model presented here has been developed based on a comprehensive and systematic review of previous literature in the field of consumer behaviour and local marketing. Schematically represented in Fig. 1, the advanced model suggests that attitude towards local products plays a fundamental role as a mediating variable in the dynamics between the intention to consume these products (dependent variable) and two key determinants: consumer ethnocentrism and identity with place (independent variables). The model also introduces a moderating variable which is the type of local product. This moderation implies that the strength and direction of the relationships mediated by attitudes towards products may vary depending on the product category, such as wines or cheeses.

Additionally, the model proposes that there are direct and positive effects that flow both from consumer ethnocentrism towards the intention to consume local products and from attitudes towards local products towards the same intention to consume. These direct effects suggest that, beyond mediation, there are other ways in which cultural values and perceptions impact consumption decisions.

Finally, we analyse how place identity directly influences both attitudes towards local products and consumption intentions, shaping a sequence of effects that culminate in specific purchase behaviours. This holistic approach provides an in-depth and detailed view of how cultural and psychological factors intertwine to shape consumption preferences in local settings, highlighting the complexity and multidimensionality of consumption decisions in globalised markets.

## 3 Materials and methods

To meet the proposed objectives, we conducted two self-administered surveys using an Internet panel in the second half of 2020. We chose this type of panel because of its efficiency in gathering data, which is due to the extensive availability of the Internet to a range of demographic groups and its cost-effectiveness [126]. It has also been demonstrated that the representativeness of data obtained on the Internet is comparable to data from probabilistic samples of the general population [127].

The group of respondents was formed by members of the general population of the Canary Islands (Spain) who were listed in the databases of a company that specialises in such panels. Certain criteria were established for participant selection: they had to be over 18 years of age (legal age to consume alcohol) and habitual consumers of cheese or wine. The frequency of consumption was defined as at least once every 15 days.

After applying these criteria in a detailed selection process to guarantee the suitability of the participants, we were able to consolidate a valid sample of 1325 individuals. From this total, 523 completed the survey related to wine, whereas 802 focused on cheese. All of the participants who completed the survey were compensated with the incentives offered by the panel company. Said company was also responsible for ensuring the quality of the responses using previously established procedures.

The surveys were divided into four main sections: filter questions and consumption habits relating to the product in question; intention and related premises; a detailed profile of the consumer; and, finally, classification data. We also included control questions to ensure that the individuals were paying due attention when completing the survey. Furthermore, we established response acceptance criteria based on the time taken to complete the survey, rejecting any that were completed in under 6 min.

The constructs were measured using a response format of a seven-point Likert scale ranging from 1 (totally disagree) to 7 (totally agree). The consumer ethnocentrism construct was evaluated using an adapted version of the CETSCALE proposed by Shimp & Sharma [63]. To measure place identity, we used criteria inspired by the work of Obst et al. [128]. In terms of attitude towards local products and intention to consume them, we adapted the items used in the studies by Zhang et al. [129] and Maksan et al. [75]. All of the constructs that comprise the proposed theoretical moderated mediation model are listed in Table 2.

Table 1 shows the demographic profile of the participants, differentiating between those who responded to the survey about wine and those who completed the cheese survey. This distribution provides a diverse picture of wine and cheese consumers based on gender, age and income level. Distribution of the respondents by gender revealed an almost equal participation of males and females in both groups. In terms of age, wine consumers were mainly concentrated in the 25-to-44-year age range, whereas cheese was most popular among those between the ages of 45 and 55. As far as income level was concerned, most of the respondents for both products were in the average income bracket.

**Table 1:** Sample profile.

	Wine consumers	Cheese consumers
Gender		
Female	46.3 %	53.1 %
Male	53.7 %	46.9 %
**Age**		
18–24 years	10.9 %	12.6 %
25–34 years	26.8 %	14.8 %
35–44 years	27.5 %	15.4 %
45–54 years	20.1 %	25.7 %
≥55 years	14.7 %	31.5 %
**Income level**		
Below average	19.4 %	23.1 %
Average	59.5 %	59.9 %
Above average	21.1 %	17.0 %
**Sample size**	523	802
**Accumulated sample size**	1325

**Table 2:** Items of the measurement model, descriptive analysis and results of the evaluation of the wine consumer group.

		Wine consumers				
*Constructs*		Mean	Standard Deviation	Loading	Composite Reliability	Average Variance Extracted
CE	Consumer ethnocentrism				0.874	0.663
CE1	Residents should always consume local products rather than imports.	4.79	1.529	0.845		
CE2	There should be very little trade of foreign products, except where necessary.	4.39	1.481	0.688		
CE3	It may cost me more in the long term, but I prefer to support local products.	5.30	1.250	0.896		
**PI**	**Place identity**				**0.901**	**0.804**
PI1	I feel that I belong here and that it is part of my identity.	5.66	1.351	0.922		
PI2	I like living here and I feel connected to the place.	5.75	1.284	0.922		
PI3	It is the best place in which to do the things I enjoy.	5.53	1.369	0.843		
**AT**	**Attitude towards local products**				**0.883**	**0.807**
AT1	I find regularly consuming local wine satisfying.	5.28	1.328	0.912		
AT2	Regularly consuming local wine gives me a feeling of positivity.	5.25	1.287	0.885		
AT3	I feel proud to consume local wine.	5.56	1.275	0.897		
**IN**	**Intention to consume local products**				**0.912**	**0.850**
IN1	I intend to consume local wine regularly.	5.25	1.361	0.926		
IN2	I have decided to consume local wine.	5.40	1.393	0.917		
IN3	I will probably consume local wine regularly.	5.29	1.378	0.922		

To verify the appropriateness of our sample size for the proposed analyses, G*Power [130] was used. This tool provided a statistical assessment that allowed us to determine whether our sample size was sufficient to effectively address our research objectives. According to the results obtained, a minimum of 280 participants were required in each of the two samples to evaluate the proposed model, which includes four variables, with a power of 95 %. We can confidently confirm that the samples used in this study greatly exceeded the requirements.

To examine the proposed conceptual model of moderated mediation and test the hypotheses, we applied the commonly used Partial Least Squares Structural Equation Modelling technique, known as PLS-SEM, using SmartPLS v.4.09.6 software [131]. PLS-SEM stands out for its ability to analyse hypotheses by estimating relationships between independent and dependent variables in a structural model [132]. In social sciences, it has gained recognition due to its ability to evaluate structural models and relationships without the need to make assumptions about the distribution of the data [58]. A distinctive element of PLS-SEM is its causal-predictive approach to statistical model analysis, seeking to provide causal explanations in the underlying structures [133].

Regarding the overall model fit, the standardised SRMR (Standardised Root Mean Square Residual) measure was applied and adapted for PLS-SEM [134]. This metric was used to ensure the absence of multicollinearity between constructs. Multicollinearity refers to the high correlation between two or more independent variables in a statistical model, which can make it difficult to identify the individual contributions of each variable to the model. In this context, checking for the absence of multicollinearity ensures that the variables in the study are not too interrelated, which could affect the reliability of the results and the interpretation of the relationships between them.

An analysis was also conducted to test the reliability and validity of the variables in the model, and the effectiveness of the model was assessed by determining the coefficient of determination (R^2^) and examining the coefficients of the relationships between the variables. The R^2^ statistic indicates how much of the variability of a specific variable is explained by the variables that predict it in a model.

Finally, to understand how the type of product moderates the relationships proposed in the structural model (research questions RQ1, RQ2 and RQ3), we performed a multi-group analysis (MGA). This technique, performed with SmartPLS v.4.09.6 software [131], helps us to understand whether the relationships between variables are consistent across different product categories.

## 4 Results

### 4.1 Descriptive analysis

The results of the descriptive analysis, which include the mean and the standard deviation of the items corresponding to the constructs of the proposed model, are shown in Table 2 for the wine consumers and in Table 3 for the cheese consumers.

**Table 3:** Items of the measurement model, descriptive analysis and results of the evaluation of the cheese consumer group.

		Cheese consumers				
*Constructs*		Mean	Standard Deviation	Loading	Composite Reliability	Average Variance Extracted
CE	Consumer ethnocentrism				0.761	0.583
CE1	Residents should always consume local products rather than imports.	4.53	1.772	0.808		
CE2	There should be very little trade of foreign products, except where necessary.	3.56	1.749	0.606		
CE3	It may cost me more in the long term, but I prefer to support local products.	5.55	1.276	0.855		
**PI**	**Place identity**				**0.874**	**0.778**
PI1	I feel that I belong here and that it is part of my identity.	6.08	1.167	0.904		
PI2	I like living here and I feel connected to the place.	6.19	1.093	0.919		
PI3	It is the best place in which to do the things I enjoy.	5.74	1.343	0.820		
**AT**	**Attitude towards local products**				**0.810**	**0.717**
AT1	I find regularly consuming local cheese satisfying.	5.55	1.326	0.883		
AT2	Regularly consuming local cheese gives me a feeling of positivity.	5.13	1.380	0.819		
AT3	I feel proud to consume local cheese.	5.82	1.266	0.836		
**IN**	**Intention to consume local products**				**0.906**	**0.841**
IN1	I intend to consume local cheese regularly.	5.63	1.275	0.923		
IN2	I have decided to consume local cheese.	5.71	1.273	0.911		
IN3	I will probably consume local cheese regularly.	5.59	1.309	0.917		

These results are expressed on a scale from 1 to 7. For the consumer ethnocentrism construct, the items exhibit scores above the centre of the scale. In the case of wine, the scores range from 4.39 to 5.30, whereas the cheese scores range from 3.56 to 5.30. In the place identity construct, the items have mean scores above the centre of the scale. These scores are more pronounced for cheese, ranging from 5.74 to 6.19 than for wine, where they range from 5.53 to 5.75. Regarding attitude towards local products, the scores of the items are almost identical in both cases, ranging from 5.25 to 5.56 for wine and 5.13 to 5.82 for cheese – well above the centre of the scale. Finally, in terms of intention to consume local products, the items are also well above the centre of the scale, with scores ranging from 5.25 to 5.40 for wine and from 5.59 to 5.71 for cheese.

These results indicate that the items of each construct are consistently above the centre of the scale for both products (wine and cheese). This suggests a certain positive trend or perception of what these constructs represent.

### 4.2 Evaluation of the global model

The analysis revealed that the SRMR fit score is 0.07 for the cheese model and 0.08 for the wine model. These scores are considered to be acceptable for the PLS-SEM method as they do not exceed the recommended cut-off value of 0.08, as mentioned by Henseler et al. [134]. Furthermore, we confirmed that there were no signs of multicollinearity between the predictive variables of the endogenous constructs as all the Variance Inflation Factor (VIF) scores were below 3.

### 4.3 Evaluation of the measurement model

We evaluated the reliability of the individual indicators of the constructs by analysing the factor loadings (λ) of the indicators about their respective constructs. As Table 2, Table 3 show, most of the loadings of the items in the measurement models exceed the cut-off value of 0.707 or are close to it [135]. Moreover, these tables also show the reliability of the constructs and it is clear that all the composite reliability scores [136] exceed the minimum reference value of 0.70 [137]. The Average Variance Extracted (AVE) measurements for all the latent variables exceed the cut-off value of 0.5, as established by Fornell & Larcker [137], which indicates that they display convergent validity.

The results in Table 4 support the discriminant validity of the constructs evaluated by meeting the Heterotrait-Monotrait ratio of correlations (HTMT) requirement (values below 0.85, [138]. Therefore, we consider the measurement model to be satisfactory and to provide solid evidence in terms of reliability, convergent validity and discriminant validity.

**Table 4:** Discriminant validity. Heterotrait-Monotrait Ratio (HTMT).

	Wine consumers				Cheese consumers			
Constructs	AT	CE	IN	PI	AT	CE	IN	PI
AT								
ET	0.601				0.561			
IN	0.641	0.508			0.780	0.572		
PI	0.551	0.392	0.445		0.551	0.468	0.550	

### 4.4 Evaluation of the structural model

The path coefficients represent the estimates of the relationships in the structural model. To evaluate its statistical significance, we applied the bootstrapping method [139]. This process involved creating 10,000 subsamples and subsequently performing a one-tailed Student's t-distribution test with n-1 degrees of freedom. We also analysed the confidence intervals [140] to gain a more complete understanding of the robustness of the results.

Table 5 summarises the mediating effects of the variables in the model. Whereas Table 6 shows specific data for wine consumers, Table 7 focuses on cheese consumers. In all three scenarios, the direct relationships between the variables are positive and significant. Specifically, attitude towards local products has the most significant direct effect on the intention to consume local products, both in the general sample (Hb: β = 0.445) and in that of the cheese consumers (Hb: β = 0.485). In the wine sample, the relationship with the greatest direct effect is between consumer ethnocentrism and attitude towards local products (Ha1: β = 0.412). However, attitude towards the consumption of local products obtained a similar score (Hb: β = 0.399). The relationship between place identity and attitude towards local products also stands out in all the scenarios.

**Table 5:** Summary of the mediating effects in the general sample.

			General sample						
			Coefficient		Bootstrap 90 % CI				
					Percentile		Bias Corrected		VAF
**CE**	H1c′	CE - > IN	0.205	sig	0.153	0.255	0.153	0.255	
	Ha1	CE - > AT	0.376	sig	0.333	0.421	0.331	0.419	
	Hb	AT - > IN	0.445	sig	0.395	0.492	0.398	0.495	
	H1med = a1*b1	CE - > AT-- > IN	0.167	sig	0.142	0.194	0.142	0.194	44.9 %
**PI**	H2c′	PI - > IN	0.170	sig	0.126	0.215	0.125	0.214	
	Ha2	PI - > AT	0.335	sig	0.286	0.384	0.288	0.386	
	Hb	AT - > IN	0.445	sig	0.395	0.492	0.398	0.495	
	H2med = a2*b2	PI - > AT - > IN	0.149	sig	0.123	0.172	0.124	0.174	46.7 %

**Table 6:** Summary of the mediating effects in the wine consumer sample.

			Wine consumers						
			Coefficient		Bootstrap 90 % CI				
					Percentile		Bias Corrected		VAF
**CE**	H1c′	CE - > IN	0.207	sig	0.110	0.296	0.112	0.298	
	Ha1	CE - > AT	0.412	sig	0.339	0.485	0.341	0.487	
	Hb	AT - > IN	0.399	sig	0.317	0.483	0.315	0.481	
	H1med = a1*b1	CE - > AT-- > IN	0.164	sig	0.123	0.209	0.122	0.209	44.3 %
**PI**	H2c′	PI - > IN	0.134	sig	0.070	0.201	0.068	0.199	
	Ha2	PI - > AT	0.344	sig	0.265	0.423	0.265	0.423	
	Hb	AT - > IN	0.399	sig	0.317	0.483	0.315	0.481	
	H2med = a2*b2	PI - > AT - > IN	0.137	sig	0.099	0.181	0.099	0.180	50.6 %

**Table 7:** Summary of the mediating effects in the cheese consumer sample.

			Cheese consumers						
			Coefficient		Bootstrap 90 % CI				
					Percentile		Bias Corrected		VAF
**CE**	H1c′	CE - > IN	0.210	sig	0.158	0.263	0.158	0.263	
	Ha1	CE - > AT	0.346	sig	0.293	0.401	0.291	0.399	
	Hb	AT - > IN	0.485	sig	0.428	0.539	0.429	0.540	
	H1med = a1*b1	CE - > AT-- > IN	0.168	sig	0.137	0.203	0.137	0.203	44.4 %
**PI**	H2c′	PI - > IN	0.180	sig	0.115	0.243	0.114	0.242	
	Ha2	PI - > AT	0.323	sig	0.256	0.384	0.258	0.386	
	Hb	AT - > IN	0.485	sig	0.428	0.539	0.429	0.540	
	H2med = a2*b2	PI - > AT - > IN	0.157	sig	0.124	0.189	0.125	0.190	46.5 %

Regarding the mediating role of attitude towards local products in the relationships that consumer ethnocentrism and place identity have with the intention to consume local products, upon analysing the percentage of Variance Accounted For (VAF) we confirmed that there was a partial mediation in both cases. According to Hair et al. [141], to confirm a total mediation, the VAF must exceed 80 %. On the other hand, a score lower than 20 % suggests insignificant mediation. In the samples analysed, the VAF was in the range of 44.3–50.6 %, which confirms partial mediation between the variables mentioned.

Table 8 reveals that the type of local product, be it wine or cheese, does not have a moderating effect on the relationships studied.

**Table 8:** Summary of the moderating effects of the type of local product.

		Path Coefficients					Multigroup analysis (MGA)		
Type of product moderation		Local wine	Sig.^a^	Local cheese	Sig.^a^	Difference	Henseler's MGA	Permutation test	Supported
ATT - > INT		0.399	***	0.485	***	0.086	ns	ns	No/No
ETH - > ATT		0.412	***	0.346	***	−0.066	ns	ns	No/No
ETH - > INT		0.207	***	0.210	***	0.003	ns	ns	No/No
PI - > ATT		0.344	***	0.323	***	−0.021	ns	ns	No/No
PI - > INT		0.134	***	0.180	***	0.046	ns	ns	No/No

a Significance level: ***p < 0.001; **p < 0.01; *p < 0.05; ns (no significance).

## 5 Discussion

This study conclusively supports all our proposed hypotheses. We highlight a significant positive relationship between consumer ethnocentrism and consumer attitudes towards local products. This finding is in line with previous research by García-Gallego et al. [73], Jianlin et al. [74], Maksan et al. [75], Miftari et al. [43], Petek et al. [76], Van Loo et al. [77] and Yildiz et al. [26], who have observed a similar relationship in cases focusing on specific products such as locally sourced wines and cheeses. Likewise, the connection between consumer ethnocentrism and intention to consume local products is confirmed, coinciding with previous research by Fernández-Ferrín et al. [78] and Nguyen et al. [79].

In parallel, place identity emerges as a significant determinant in the formation of favourable attitudes towards local products, supporting the findings of Yildiz et al. [26], Zeugner-Roth et al. [27] and Zhang & Khare [96]. Furthermore, consumers who demonstrate a strong place identity tend to consume more products from their region, corroborating the findings of Fusté-Forné [30] and Nguyen et al. [97].

The results of this study also confirm the direct relationship between attitude towards local products and intention to consume them. This pattern is reaffirmed by authors like Achabou et al. [100], Hussain et al. [101], Kol et al. [102] and Skallerud & Wien [50], as well as by specialised studies of wine and cheese, such as those of Gultek et al. [103], Kolyesnikova et al. [104] and Torres-Salas et al. [105].

However, one of the most valuable contributions of this study to the field of consumer behaviour, particularly regarding local products, consists of the mediated relationships that we have identified. By identifying a partial mediation of attitude towards local products in the connection between consumer ethnocentrism and consumption intention, we add an innovative and solid perspective to the existing literature. This finding insinuates that attitude towards local products is configured as a determining factor in the formation of consumption intention, a finding that has resonated in previous studies such as those of Chaturvedi et al. [65], Jianlin et al. [74] and Maksan et al. [75].

We also highlight the partial mediation of attitude towards local products in the relationship between place identity and consumption intention. This confirmation not only fills a gap in the current literature but also paves the way for future research work. Fundamentally, we have deduced that attitude towards local products operates as a key mediating variable that has a decisive influence on the generation of consumption intention. This discovery has important implications both for designing marketing strategies and for product development.

Regarding the exploratory questions expressed as research questions, we determined that the type of local product does not lead to significant differences in the mediated or direct relationships analysed. These results concur with those of Nguyen et al. [79], who did not find any differences in the impacts of consumer ethnocentrism on intention to purchase local products according to the type of product, and those of Yang et al. [125], who did not detect any differences in the relationships between local/global identity and purchase intention according to the type of product. Likewise, in terms of the relationship between attitude towards local products and intention to consume them, no variations were found based on the type of product.

## 6 Conclusions

In this study, we have devised and validated a moderately mediated explanatory model. The findings represent a valuable starting point for understanding the relationships between consumer ethnocentrism, place identity, attitude towards local products and consumption intention. The mediating role of attitude towards local products is confirmed, together with the absence of significant differences in consumer response according to the specific type of local product, exemplified by wines and cheeses in our study context, the Canary Islands.

Promoting the consumption of local products proves to be a fundamental strategy to strengthen regional economies, foster sustainability and preserve cultural identity. This research provides us with a clearer picture of the crucial influence of consumer ethnocentrism and place identity on attitudes and purchasing behaviour related to local products.

Attitude towards local products proves to be a significant mediator between consumer ethnocentrism and consumption intention, as well as between place identity and consumption intention. These findings underline the importance of consumer perceptions and attitudes towards local products in purchasing decisions. It is essential to recognise that, despite the diversity of products studied, no significant differences were identified according to the specific type of local product, suggesting a universality of attitudes and behaviours associated with these products in the study region, the Canary Islands.

In an increasingly globalised world in which consumers can choose from a plethora of products from around the world, it is vital to understand the underlying motivations behind the consumption of local products. Marketing strategies need to consider the emotional and cultural connection that consumers establish with local products, boosting the added value that these products represent in terms of identity and belonging.

In this sense, we hope that our research will serve as a starting point for future studies that further explore the dynamics of local consumption in various contexts and product categories. These findings could be effectively applied to boost local economies and encourage sustainable consumption. A better understanding of the complexities of consumer behaviour at the local level paves the way for targeted policies and strategies to promote economic development and sustainability in local communities.

### 6.1 Practical implications

The findings of this study shed light for companies, organisations and public policies on the strategic relevance of promoting the consumption of local products, not only as a boost for regional economies but also as a sustainable and culturally rooted way forward. In light of a global environment marked by uncertainty, ranging from climate policy and armed conflicts to the COVID-19 pandemic and currency fluctuations, the practical implications must be framed with these challenges in mind.

First, it highlights the need for companies to adapt their marketing strategies to highlight the local origin of their products. This adaptation not only reinforces the perception of what is ‘local’ but also strengthens consumers' affinity with these products and stimulates their purchase intention. In times of uncertainty, where emotional and cultural connection becomes even more important, these strategies can provide stability and differentiation.

Collaboration between businesses and local authorities proves to be a crucial tool to cultivate an environment that promotes and celebrates local pride. This joint effort can be manifested through campaigns, events and activities that highlight the value of consuming regional products, acting as a response to uncertainty and strengthening the local social and economic fabric.

It is essential to recognise that, to maximise the intention to consume local products, one must not only enhance consumer ethnocentrism and place identity but also cultivate a positive attitude towards these products. Key strategies include educating consumers about the inherent benefits of opting for local products, providing transparent information about their origin and production process, and ensuring quality and consistency in every purchase. Emotional and cultural connections become even more important in the context of global uncertainty. Establishing loyalty programmes and forming emotional connections with consumers are crucial steps for building favourable attitudes towards local products, and act as an anchor in times of instability.

Collaboration between producers and administrations can be extended to events that allow consumers, especially the younger generations, to directly appreciate the quality and uniqueness of local products. These initiatives not only boost the local economy through direct sales but also contribute to shaping positive perceptions that influence predisposition towards local produce.

Strategic alliances with other local businesses and organisations can be essential, creating a collective identity and sense of community as far as regional products are concerned. Events, fairs or markets dedicated to these products can increase their visibility and attractiveness to consumers.

Finally, although no differences based on product type have been identified in this study, companies must understand the specific preferences of their consumers and educate them about the benefits of consuming local products, addressing not only economic but also environmental and social aspects. Synergies between local companies can enhance these efforts, benefiting the whole community in a global environment marked by uncertainty.

### 6.2 Limitations and future research directions

It is necessary to mention some limitations that should be considered when interpreting the results of our study. Our study was conducted specifically in the Canary Islands, which may limit the generalisation of the results to other regions or countries with different cultures and economies. To address this question, future research could explore cross-cultural comparison studies, shedding light on how variations in cultural perspectives may shape consumer choices.

Furthermore, even though we focused on wine and cheese and we did not see any differences based on the type of product, extrapolation to other categories of local products may not be direct. It is therefore recommended that further research should explore these possible variations between different categories of local products.

Finally, it is important to take into account that consumer behaviour may vary over time due to market trends, global events and other influencing factors which may affect the ongoing applicability of the results. In this sense, we suggest that longitudinal studies be considered in the future. This approach would allow for a more complete understanding of how attitudes and behaviours towards local products may change and adapt over time.

## Data availability statement

Data associated with your study has not been deposited into a publicly available repository. Data will be made available on request.

## Funding statement

No external funding to report.

## CRediT authorship contribution statement

**Edgar J. Sabina del Castillo:** Writing – review & editing, Writing – original draft, Visualization, Validation, Supervision, Software, Methodology, Investigation, Funding acquisition, Formal analysis, Data curation, Conceptualization. **Ricardo J. Díaz Armas:** Writing – original draft, Validation, Supervision, Methodology, Investigation, Conceptualization. **Desiderio Gutiérrez Taño:** Writing – original draft, Validation, Software, Methodology, Investigation, Data curation.

## Declaration of competing interest

The authors declare that they have no known competing financial interests or personal relationships that could have appeared to influence the work reported in this paper.
